# Pituitary Adenoma Nitroproteomics: Current Status and Perspectives

**DOI:** 10.1155/2013/580710

**Published:** 2013-03-07

**Authors:** Xianquan Zhan, Xiaowei Wang, Dominic M. Desiderio

**Affiliations:** ^1^Key Laboratory of Cancer Proteomics of Chinese Ministry of Health, Xiangya Hospital, Central South University, 87 Xiangya Road, Changsha, Hunan 410008, China; ^2^Hunan Engineering Laboratory for Structural Biology and Drug Design, Xiangya Hospital, Central South University, 87 Xiangya Road, Changsha, Hunan 410008, China; ^3^State Local Joint Engineering Laboratory for Anticancer Drugs, Xiangya Hospital, Central South University, 87 Xiangya Road, Changsha, Hunan 410008, China; ^4^The Charles B. Stout Neuroscience Mass Spectrometry Laboratory, Department of Neurology, College of Medicine, University of Tennessee Health Science Center, 847 Monroe Avenue, Memphis, TN 38163, USA

## Abstract

Oxidative stress is extensively associated with tumorigenesis. A series of studies on stable tyrosine nitration as a marker of oxidative damage were performed in human pituitary and adenoma. This paper reviews published research on the mass spectrometry characteristics of nitropeptides and nitroproteomics of pituitary controls and adenomas. The methodology used for nitroproteomics, the current status of human pituitary nitroproteomics studies, and the future perspectives are reviewed. Enrichment of those low-abundance endogenous nitroproteins from human tissues or body fluid samples is the first important step for nitroproteomics studies. Mass spectrometry is the essential approach to determine the amino acid sequence and locate the nitrotyrosine sites. Bioinformatics analyses, including protein domain and motif analyses, are needed to locate the nitrotyrosine site within the corresponding protein domains/motifs. Systems biology techniques, including pathway analysis, are necessary to discover signaling pathway networks involving nitroproteins from the systematically global point of view. Future quantitative nitroproteomics will discover pituitary adenoma-specific nitroprotein(s). Structural biology techniques such as X-ray crystallography analysis will solidly clarify the effects of tyrosine nitration on structure and functions of a protein. Those studies will eventually address the mechanisms and biological functions of tyrosine nitration in pituitary tumorigenesis and will discover nitroprotein biomarkers for pituitary adenomas and targets for drug design for pituitary adenoma therapy.

## 1. Introduction

Nitration of a tyrosine residue (NO_2_-Tyr-Prot) in a protein generated from the primary *in vivo* peroxynitrite pathway, as well as the secondary myeloperoxidase and other metalloperoxidase reaction pathways [1, 2], is a potential marker of oxidative/nitrosative injuries [3, 4] and might play important molecular roles in human pituitary physiology and pathology. The pituitary plays central regulation roles in the hypothalamic-pituitary-target organ axis systems. Studies have indicated that nitric oxide (NO) [3] and nitric oxide synthase (NOS), which are the upstream molecules that promote formation of tyrosine nitration, participate in multiple axis systems [5–7]: growth hormone (GH) [8–10], prolactin (PRL) [11], adrenocorticotropin (ACTH) [12], follicle-stimulating hormone (FSH) [13], and luteinizing hormone (LH) [7, 13–15]. A global proteomics approach was used to investigate protein tyrosine nitration in human pituitary and adenoma tissues, namely, nitroproteomics. A total of eight nitrotyrosine-containing proteins (nitroproteins) in a human pituitary postmortem tissue and nine nitroproteins and three nitroprotein-interacting proteins in a human nonfunctional adenoma tissue [3, 4, 16] were identified with tandem mass spectrometry (MS/MS). Nitrotyrosine sites located within the important protein domains or motifs [4] were involved in the tumor biological characteristics [4]. 

 The detection and identification of endogenous nitroproteins is very challenging because of its very low level (one nitroprotein per ~10^6^ proteins) in a proteome [17, 18]. Antinitrotyrosine antibody-based enzyme-linked immunosorbent assay (ELISA) [2, 19], immunoprecipitation [4], and one/two-dimensional-gel-electrophoresis- (1DGE/2DGE-) based Western blot analyses [3, 16, 20, 21] are effective approaches to detect and preferentially enrich endogenous nitroproteins. ELISA can measure the nitrotyrosine content, and an ELISA assay kit is commercially available (Upstate Catalog no. 17-136). 1DGE/2DGE-based Western blots can separate and preferentially enrich endogenous nitroproteins and also determine the relative level of nitrotyrosine. Immunoprecipitation can preferentially enrich endogenous nitroproteins from a complex proteome for mass spectrometry analysis. Tandem mass spectrometry (MS/MS) can identify a nitrotyrosine site in a nitroprotein [3, 4, 16, 22]. However, the sensitivity (generally high femtomole to low picomole) of mass spectrometry is the bottleneck due to the low abundance of nitroproteins in a complex pituitary proteome. Thus, preferential enrichment of endogenous nitroproteins or nitropeptides is an essential step prior to MS/MS analysis [22]. For human pituitary nitroproteomics studies, 2DGE-based nitrotyrosine Western blot analysis [3, 16] and nitrotyrosine immunoaffinity enrichment [4] were used to separate and preferentially enrich endogenous nitroproteins from a complex human pituitary control and adenoma tissue. Enriched nitroproteins were subject to trypsin digestion, followed by the amino acid sequence analysis with MS/MS to identify nitroprotein and nitrotyrosine sites. Bioinformatics was used to determine structural/functional domains and motifs of a nitroprotein and to locate the nitrotyrosine site within a protein domain/motif to clarify the roles of tyrosine nitration in a protein [4]. Pathway analysis-based systems biology was used to discover the pathway networks that involved endogenous nitroproteins from a systematical and comprehensive angle [23]. In addition, mass spectrometry characteristics of the standard nitropeptides [24] were analyzed to obtain the fragmentation to assist in the interpretation of the mass spectrometry spectrum of a tryptic peptide derived from an endogenous nitroprotein in a proteome.

## 2. Biological Roles of Oxidative/Nitrative Stresses in Pituitary Adenoma Pathophysiology

Reactive-oxygen/nitrogen-species- (ROS/RNS-) mediated oxidative/nitrative stresses play important roles in cellular, physiological and pathological processes [24–27]. ROS are formed by several mechanisms [1, 25, 28], including (i) synthesis through dedicated enzymes such as NADPH oxidase and myeloperoxidase, (ii) interaction of ionizing radiation with biological molecules, and (iii) an unavoidable byproduct of cellular respiration. Electrons from the electron transport chain leak away from the main path such as ubiquinone to reduce oxygen molecules to the superoxide anion. RNS are a family of antimicrobial molecules generated from the nitric oxide radical (^•^NO) and superoxide anion (O_2_^−•^) produced through the enzymatic activity of inducible nitric oxide synthase (iNOS), endothelial nitric oxide synthase (eNOS) neuronal nitric oxide synthase (nNOS), and NADPH oxidase, respectively [3, 4, 22, 29]. iNOS is expressed primarily in many cell types such as macrophages after induction by cytokines and microbial products, notably interferon-gamma (IFN-*γ*) and lipopolysaccharide (LPS), or during pathology and disease [28, 30]. RNS act together with ROS to damage cells to cause nitrosative stress. Thus, these two species are often collectively called as ROS/RNS. RNS are produced in animals from the reaction of nitric oxide (^•^NO) and superoxide anion (O_2_^−•^) to form the more toxic peroxynitrite (ONOO^−^) [17, 31].

Specific amino acid residues in a protein are sensitive targets that can be modified by ROS/RNS [24]. Tyrosine nitration in a protein is an important redox-related modification that derives from, not only the main *in vivo* peroxynitrite pathway, but also myeloperoxidase and other metalloperoxidase reaction pathways [1, 2, 22]. Protein tyrosine nitration involves addition of an electron-withdrawing group, -NO_2_, to the phenolic ring of the tyrosine residue [4] to decrease the electron density of the phenolic ring of a tyrosine residue in a protein to change the phenolic pKa value (from ~10 for tyrosine) into the physiological pH range (~7.1 for 3-nitrotyrosine) to affect chemical properties of a tyrosine residue [4, 32, 33]. If the nitration occurred within the interacting region between an enzyme and its substrate and/or between a receptor and its ligand, then the decreased electron density could impact on the interaction intensity (enzyme-substrate, receptor-ligand) to affect the functions of that protein [4]. Furthermore, some studies demonstrated that biological protein nitration might be a dynamic and reversible process between nitration and denitration due to a discovery of a putative denitrase [33–35]. Thus, protein tyrosine nitration might have biological consequences such as redox signaling and neurotransmission in addition to its pathological consequences. Also, tyrosine nitration would compete with phosphorylation of a tyrosine residue because tyrosine nitration occurs within a tyrosine phosphorylation motif ([R or K]-x2(3)-[D or E]-x3(2)-[Y]) [36–38]. Therefore, protein tyrosine nitration occurs under physiological conditions, is enhanced under pathological conditions, and might be reversed by enzymatic or nonenzymatic mechanisms [22]. This modification can alter the functions of a protein and is associated with many physiological/pathological processes such as inflammatory diseases, neurodegenerative diseases, and tumors [3, 4, 17, 21, 27, 31].

ROS/RNS are involved in multiple hypothalamic-pituitary-target organ axis systems and are elevated in pituitary tumors [3, 4, 16, 22]. NOS is extensively expressed in the rat and human pituitary, and has an elevated activity in pituitary adenomas [3, 5–7, 39, 40]. Three types of NOS (eNOS, nNOS, and iNOS) are expressed in the pituitary gland and in pituitary adenomas, and an elevated activity of eNOS was found in the endothelial cells of pituitary adenomas [3]. nNOS and its mRNA were found to be increasing in human pituitary adenomas, and were located to the secretory and folliculostellate cells [3]. iNOS was found in rat pituitary cells that were induced by interferon-gamma (IFN-*γ*) that significantly increased NO production [41]. NO activates release of luteinizing hormone-releasing hormone (LHRH) and follicle-stimulating hormone-releasing hormone (FSHRH) from the hypothalamus and of LH and FSH from the pituitary [3, 4, 13–16, 42]. NO either participates in LH secretion in gonadotrophs or requires the participation of gonadotrophs [7]. NO might stimulate or inhibit secretion of prolactin; circulating NO changed in dopamine-treated hyperprolactinaemia patients [3, 11, 43–45]. NO regulates section of growth hormone (GH) in the normal human pituitary and in acromegaly and modulates GH secretion in a dose-dependent manner in GH adenoma cells [3, 5, 46–48]. NO plays important roles in the hypothalamic-pituitary-adrenocortical axis inhibition of ACTH release [12]. Therefore, upstream molecules (NO and NOS) that form tyrosine nitration in a protein are extensively associated with physiological and pathological processes of pituitary and are especially elevated in the pituitary adenomas [3, 4, 16]. Our global nitroproteomics data confirmed that protein tyrosine nitration existed in human pituitary postmortem tissues [3, 16] and nonfunctional pituitary adenoma tissues [4]. Those nitroproteins played important roles in the physiological and pathological processes of a human pituitary [3, 4, 16]. Therefore, ROS/RNS might be important in normal human pituitary function and relevant to dysfunction in human pituitary adenomas.

## 3. Mass Spectrometric Characteristics of Nitropeptide

 The mass spectrometry behavior of a nitrotyrosine-containing peptide (nitropeptide) greatly differs between matrix-assisted laser desorption ionization (MALDI) and electrospray ionization (ESI) [24, 49, 50]. The MALDI UV laser can induce photochemical decompositions of the nitro group (-NO_2_) to decrease the precursor-ion intensity of a nitropeptide to complicate the MS spectrum [24, 49–51]. ESI does not induce those decompositions [24, 27, 49–53]. In order to assist in the interpretation of MS identification of endogenous nitroproteins in human tissues and fluids, MALDI MS and MS/MS were used to study the fragmentation pattern of *in vitro* nitrotyrosine-containing peptides [24], including synthetic leucine enkephalin (LE1: Y-G-G-F-L, 555.1818 Da), nitro-Tyr-leucine enkephalin [LE2: (3-NO_2_)Y-G-G-F-L, 600.0909 Da], and d5-Phe-nitro-Tyr-leucine enkephalin [LE3: (3-NO_2_)Y-G-G-(d5)F-L, 605.1818 Da], with a vacuum MALDI-linear ion-trap mass spectrometer (vMALDI-LTQ).

**Figure 1:**
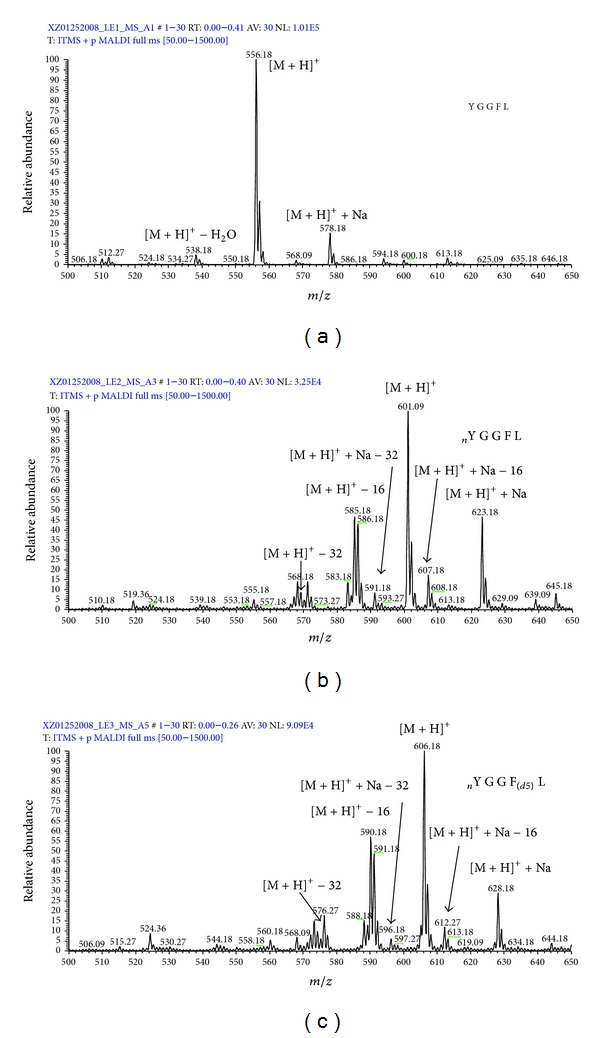
MALDI MS spectra of LE1 (a), LE2 (b), and LE3 (c). nY = nitro-Tyr. F(d5) = Phe residue with five 2H (d) atoms. Reproduced from Zhan and Desiderio (2009) [24], with permission from Elsevier Science, copyright 2009.

**Figure 2:**
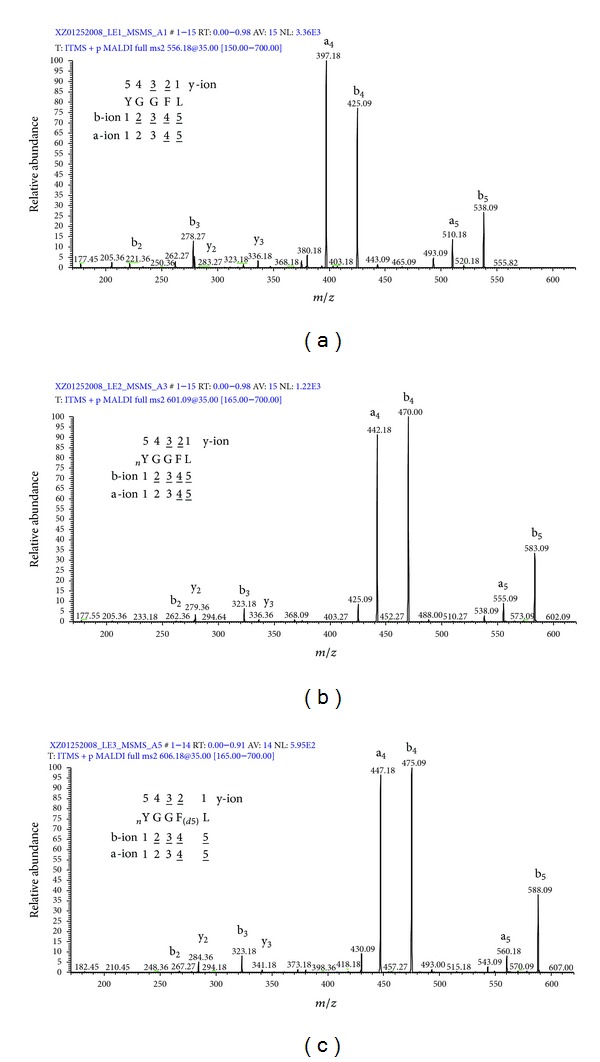
MS^2^ spectra of LE1 (a), LE2 (b), and LE3 (c). nY = nitro-Tyr. F(d5) = Phe residue with five 2H (d) atoms. Reproduced from Zhan and Desiderio (2009) [24], with permission from Elsevier Science, copyright 2009.

**Figure 3:**
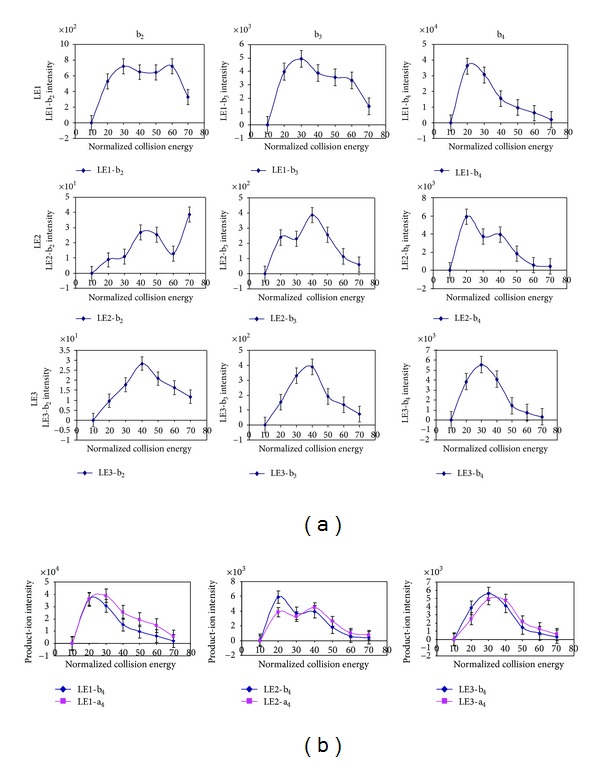
The effect of collision energy on the fragmentation of nitropeptides. (a) Relationship between collision energy and the product-ion intensity (*n* = 3). (b) Relationship between collision energy and the product-ion b_4_ and a_4_ intensities (*n* = 3). Reproduced from Zhan and Desiderio (2009) [24], with permission from Elsevier Science, copyright 2009.

The UV laser-induced photochemical decomposition (loss of one or two oxygen atoms of the nitro group to form the unique decomposition pattern of ions ([M + H]^+^ − 16 and [M + H]^+^ − 32) occurred in LE2 and LE3 compared to LE1 (Figure 1) [24]. A similar decomposition pattern ([M + H]^+^ + Na − 16 and [M + H]^+^ + Na − 32) for loss of one or two oxygen atoms occurred for the sodium adduct ([M + H]^+^ + Na) of LE2 and LE3 compared to the sodium adduct of LE1 [24]. A product ion ([M + H]^+^ − 30) was also observed in the LE2 and LE3 spectra, which could result from the reduction of the nitro group (-NO_2_) to an amino group (-NH_2_) [50]. Moreover, the base-peak intensity of the [M + H]^+^ ion of LE1 (NL = 1.01E5) was much higher than that of LE2 (NL = 3.25E4) and LE3 (NL = 9.09E4), demonstrating that photochemical decomposition decreased ion intensity and complicated the MS spectrum [24]. However, recognition of this unique decomposition pattern unambiguously identified a nitrotyrosine. For vMALDI-MS/MS analysis, b- and a-ions were the most intense fragment ions compared to the y-ions (Figure 2) [24]. Compared to the unmodified peptides (LE1), more collision energy optimized fragmentation of the nitropeptide (Figure 3(a)) but increased the intensity of the a_4_-ion and decreased the intensity of the b_4_-ion (a-ion = the loss of CO from a b-ion) (Figure 3(b)). Furthermore, optimized laser fluence maximized fragmentation of the nitropeptide. Although MS^3^ analysis confirmed the MS^2^-derived amino acid sequence, MS^3^ analysis requires a higher amount of peptides relative to MS^2^ [24]. Thus, MS^3^ analysis might not be suitable for routine analysis of endogenous low-abundance nitroproteins. Only when a target is determined, can MS^3^ be used for confirmation. To detect a nitropeptide, the amount of peptides must reach the sensitivity of a mass spectrometer; for our synthetic nitropeptides, the sensitivity of vMALDI-LTQ was 1 fmol for MS detection and 10 fmol for MS^2^ detection [24].

## 4. Enrichment of Endogenous Nitroproteins in Human Pituitary Adenomas

Nitrotyrosine formed from reaction of free or protein-bound tyrosine with RNS, such as free-radical nitrogen dioxide [54] and peroxynitrite [55], has a low-abundance (1 in ~10^6^ tyrosines) oxidative protein modification in an *in vivo* proteome [17, 18]. Moreover, mass spectrometry is the crucial approach to identify nitroproteins/nitropeptides and modified sites [3, 4, 16]; however, mass spectrometry is limited by its sensitivity, generally at the levels of high femtomole to low picomole [22]. Therefore, isolation and preferential enrichment of the nitroproteins/nitropeptides are essential prior to mass spectrometry analysis [22, 27, 52, 53]. For human pituitary adenoma nitroproteomics studies, two methods were used to isolate and preferentially enrich nitroproteins from a pituitary proteome prior to mass spectrometry, including 2DGE-based nitrotyrosine Western blotting analysis [3, 16] and nitrotyrosine-affinity-column- (NTAC-) based enrichment [4].

**Figure 4:**
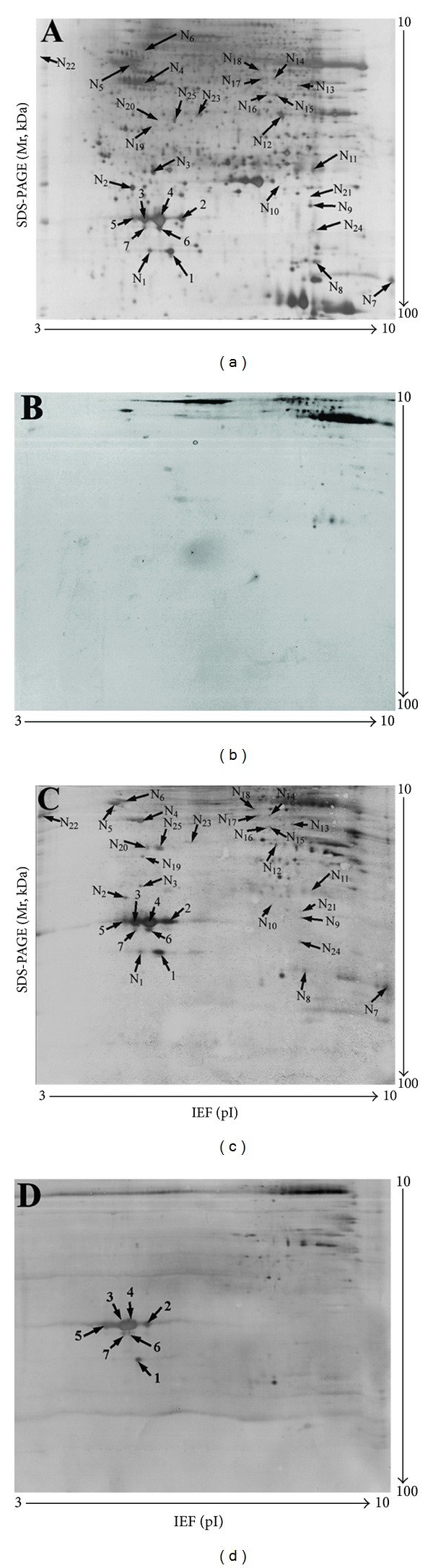
Two-dimensional Western blotting analysis of anti-3-nitrotyrosine-positive proteins in a human pituitary (70 ug protein per 2D gel). (a) Silver-stained image on a 2D gel before the transfer of proteins onto a PVDF membrane. (b) Silver-stained image on a 2D gel after the transfer of proteins onto a PVDF membrane. (c) Western blot image of anti-3-nitrotyrosine-positive proteins (anti-3-nitrotyrosine antibodies + secondary antibody). (d) Negative control of a Western blot to show the cross reaction of the secondary antibody (only the secondary antibody; no anti-3-nitrotyrosine antibody). Reproduced from Zhan and Desiderio (2007) [16], with permission from Elsevier Science, copyright 2007.

2DGE-based nitrotyrosine Western blotting [3, 16] involved proteins extracted from a postmortem control pituitary separated by isoelectric point (pI) and relative molecular weight (Mr); separated proteins transferred to a polyvinylidene fluoride (PVDF) membrane; incubation with antinitrotyrosine antibody; and visualization (Figure 4). In the silver-stained 2D gel image (pI 3–10; Mr 10–100 kDa) that contained ca. 1000 protein spots (Figure 4(a)), a total of 32 nitrotyrosine-positive Western blot spots were detected (Figure 4(c)) with 2D gel image analysis software by comparing the digitized Western blot image (Figure 4(c)) to the negative control (Figure 4(d)). Also, each nitrotyrosine-positive Western blot spot (Figure 4(c)) was matched to corresponding silver-stained 2D gel spots (Figure 4(a)) so that the silver-stained gel spots were excised for mass spectrometry analysis. Therefore, even though the abundance of a nitroprotein in a human tissue proteome is very low, 2DGE separates and enriches each nitroprotein to improve its immunodetection and MS characterization.

**Figure 5:**
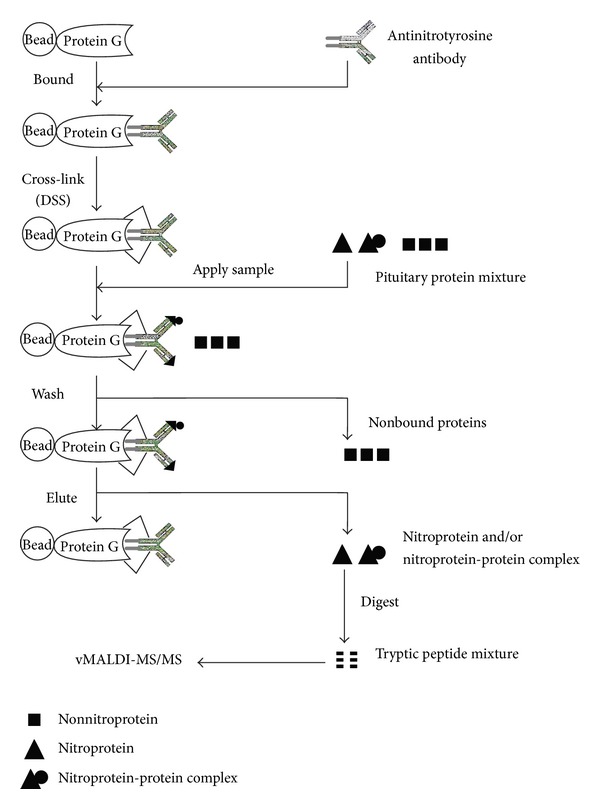
Experimental flow chart to identify nitroprotein and nitroprotein-protein complexes with NTAC-based vMALDI-LTQ MS/MS. The control experiment (without any anti-3-nitrotyrosine antibody) was performed in parallel with the NTAC-based experiments. Reproduced from Zhan and Desiderio (2006) [4], with permission from Elsevier Science, copyright 2006.

The NTAC method [4] (Figure 5) was used to preferentially enrich nitroproteins from a human pituitary adenoma proteome. Antinitrotyrosine antibodies were cross-linked to protein G beads and incubated with a pituitary adenoma protein sample. Nitroproteins and nitroprotein-protein complexes (interactomes) were bound to the cross-linked anti-nitrotyrosine antibodies. Bound nitroproteins and nitroprotein-protein complexes were eluted to provide an enriched nitroprotein sample, followed by trypsin digestion and mass spectrometry analysis. The detailed NTAC procedure was described [4]. NTAC is an effective method to isolate and enrich nitroproteins from a complicated human pituitary adenoma proteome to improve MS/MS identification of very low-abundance nitroproteins.

## 5. Tandem Mass Spectrometry Identification of Nitroproteins and Nitrotyrosine Sites in Human Pituitary and Adenoma

Tandem mass spectrometry is the essential method to obtain the amino acid sequence of a tryptic peptide or nitropeptide enzymatically digested from a nitroprotein and to determine nitrotyrosine sites [3, 4, 16]. A total of 32 2D gel spots corresponding to nitrotyrosine immunopositivity from a postmortem pituitary control tissue were excised, and proteins were extracted to identify nitroprotein and nitrotyrosine sites. Eight nitroproteins and eight nitrotyrosine sites were identified in the postmortem control pituitary (Table 1) [3, 16], including synaptosomal-associated protein, actin, immunoglobulin alpha Fc receptor, cGMP-dependent protein kinase 2, stanniocalcin 1, mitochondrial cochaperone protein HscB, progestin and adipoQ receptor family member III, and proteasome subunit alpha type 2. Those nitroproteins participate in multiple functions, including neurotransmission, cellular immunity, cellular structure and mobility, calcium and phosphate metabolism, cochaperone in iron-sulfur cluster assembly in mitochondria, membrane receptor, and the ATP/ubiquitin-dependent nonlysosomal proteolytic pathway.

**Figure 6:**
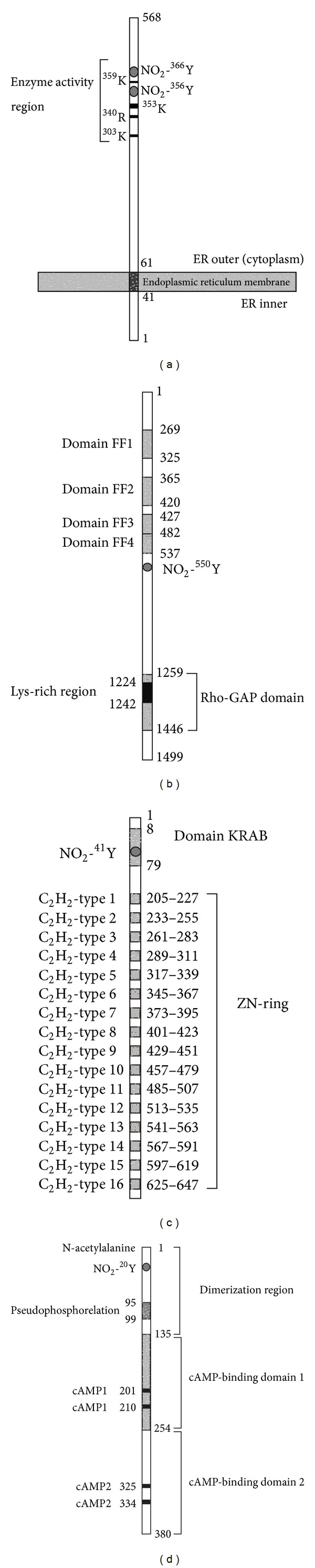
Nitration site and functional domains of four nitroproteins. (a) Sphingosine-1-phosphate lyase 1. The site 353 K is a pyridoxal phosphate-binding motif. (b) Rho-GTPase-activating protein 5. (c) Zinc finger protein 432. The KRAB domain is a transcriptional suppressor. The ZN-RING is a DNA-binding region. (d) cAMP-dependent protein kinase type I-beta regulatory subunit. Modified from Zhan and Desiderio (2006) [4] with permission from Elsevier Science and reproduced from Zhan and Deiderio (2009) [22] with permission from Springer Science.

The NTAC-based enriched nitroprotein samples from a pituitary adenoma were subjected to digestion with trypsin, followed by MS/MS analysis [4]. A total of nine nitroproteins and ten nitrotyrosine sites were identified from a pituitary adenoma tissue (Table 1; Figure 6), including sphingosine-1-phosphate lyase 1, zinc finger protein 432, cAMP-dependent protein kinase type I-beta regulatory subunit, Rho-GTPase-activating protein 5, leukocyte immunoglobulin-like receptor subfamily A member 4, centaurin-beta 1, proteasome subunit alpha type 2, interleukin 1 family member 6, and rhophilin 2. Also, three proteins including glutamate receptor-interacting protein 2, ubiquitin, and interleukin 1 receptor-associated kinase-like 2 were discovered to interact with nitroproteins (Table 1) to form three nitroprotein-protein complexes, including nitrated proteasome-ubiquitin complex, nitrated beta-subunit of cAMP-dependent protein kinase (PKA) complex, and nitrated interleukin 1 family member 6-interleukin 1 receptor-interleukin 1 receptor-associated kinase-like 2 (IL1F6-IL1R-IRAK2) [4]. 

**Figure 7:**
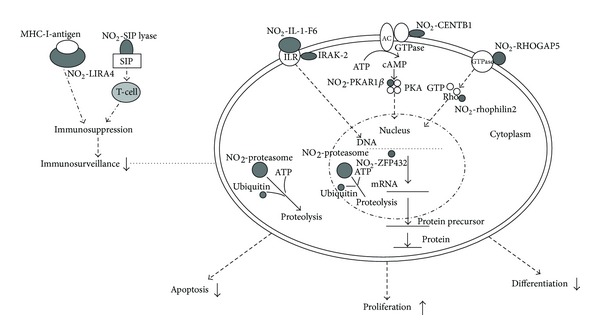
Experimental data-based model of nitroproteins and their functions in human nonfunctional pituitary adenomas. NO_2_^−^: nitroprotein. Reproduced from Zhan and Desiderio (2006) [4] with permission form Elsevier Science.

Those nine nitroproteins and three nitroprotein-protein complexes were rationalized into a corresponding functional system (Figure 7) [4]. The nitrated proteasome-ubiquitin complex is an important enzymatic complex that is involved in the intracellular nonlysosomal proteolytic pathway [4, 46, 47]. The nitrated LIRA4 might be involved in the immune system. The nitrated S1P lyase 1 participates in sphingolipid metabolism to regulate cell proliferation, survival, and cell death as well as the immune system [4, 56–58]. The nitrated CENT-beta 1 and the nitrated PKAR1-beta are involved in the PKA signal pathway. IRAK-2 in the IL1-R complex and the nitrated IL1-F6 are involved in the cytokine system. The nitrated ZFP432 is involved in transcription regulatory systems. The nitrated RHOGAP5 and the nitrated rhophilin 2 are involved in the GTPase signal pathway [4].

## 6. Bioinformatics Recognition of Nitrotyrosine-Containing Protein Domain/Motif of Nitroprotein

The specific domains or motifs in a protein would sustain corresponding intracellular biological functions. Location of nitrotyrosine sites into a protein domain or motif would benefit the accurate elucidation of the biological activities of tyrosine nitration. Protein domain analysis softwares, including ScanProsite (http://us.expasy.org/tools/scanprosite/), Motifscan (http://myhits.isb-sib.ch/cgi-bin/motif_scan), Inter-ProScan (http://www.ebi.ac.uk/InterProScan/), ProDom (http://prodom.prabi.fr/prodom/current/html/form.php), and Pfam (http://www.sanger.ac.uk/Software/Pfam/), were used to detect the statistically significant domains of each nitroprotein and to locate the identified nitrotyrosine site within a protein domain to gain insight into the effect of tyrosine nitration on the protein functions [4]. More information can be obtained on the Swiss-Prot annotation page of each protein. 

This method was used to analyze the protein domain and motif of each nitroprotein identified from human pituitary adenoma tissue [4]. An exciting result showed most nitrotyrosine sites occur within important protein domains and motifs. For example, sphingosine-1-phosphate lyase 1 (S1P lyase 1), nitrated in human pituitary adenoma [4] (Figure 6(a)), is a key enzyme to catalyze the decomposition of S1P. Two nitrations (NO_2_-^356^Y and NO_2_-^366^Y) within the enzyme activity region could decrease the interaction intensity of enzyme:substrate (S1P lyase 1:S1P) to decrease the decomposition of S1P because the nitro group (-NO_2_) is an electron-withdrawing group that could decrease the level of enzyme-substrate binding. Studies [56] have demonstrated that S1P, ceramide (Cer), and sphingosine (Sph), which are the sphingolipid metabolites, play an important role to regulate cell proliferation, survival, and cell death. Cer and Sph usually inhibit proliferation and promote apoptosis, whereas S1P stimulates growth and suppresses apoptosis. Because these metabolites are interconvertible, their relative levels determine cell fate. The nitration of S1P lyase 1 could increase the level of S1P relative to Cer and Sph, to stimulate the tumor cell proliferation and inhibit the apoptosis. Rho-GTPase-activating protein 5 (Figure 6(b)) contains four FF domains [59] and one Rho-GAP domain, and nitration (NO_2_-^550^Y) occurred within the region between two domains (FF4 and Rho-GAP) could affect Rho-GTPase signal transduction [4]. Zinc finger protein 432 (Figure 6(c)) is a transcript factor that includes 16 C_2_H_2_-type zinc fingers that bind DNA and one Kruppel-associated box (KRAB) domain that functions as a transcriptional suppressor [60–62]; nitration (NO_2_-^41^Y) within the KRAB domain could impair transcriptional suppression [4]. The cAMP-dependent protein kinase type I-beta regulatory subunit (PKAR1-beta) (Figure 6(d)) contains one N-terminal dimerization domain, one inhibitory region (pseudophosphorylation), and two cAMP-binding domains. Each cAMP-binding domain contains two cAMP-binding sites. Nitration (NO_2_-^20^Y) within the dimerization region could affect dimerization of two regulatory chains [4].

## 7. Systems Biological Analysis of Signaling Pathway Networks That Involve Nitroproteins

Systems biology is a comprehensive quantitative analysis of the manner in which all components of a biological system interact functionally over time [63–65]. Relative to the traditional molecular biology methods that had been used to study the role of a single gene, single protein, or single small-molecule model, high-throughput “-omic” technologies such as genomics, transcriptomics, proteomics, and metabolomics have driven the rapid development of systems biology to study a multiple-factor model of disease and to address the network of interaction and regulatory events that contribute to a disease [23]. Pathway biology is one important component of systems biology and is used to extensively analyze “-omic” data to mine significantly signaling pathway networks and to address the biological significance of those “-omic” data.

**Figure 8:**
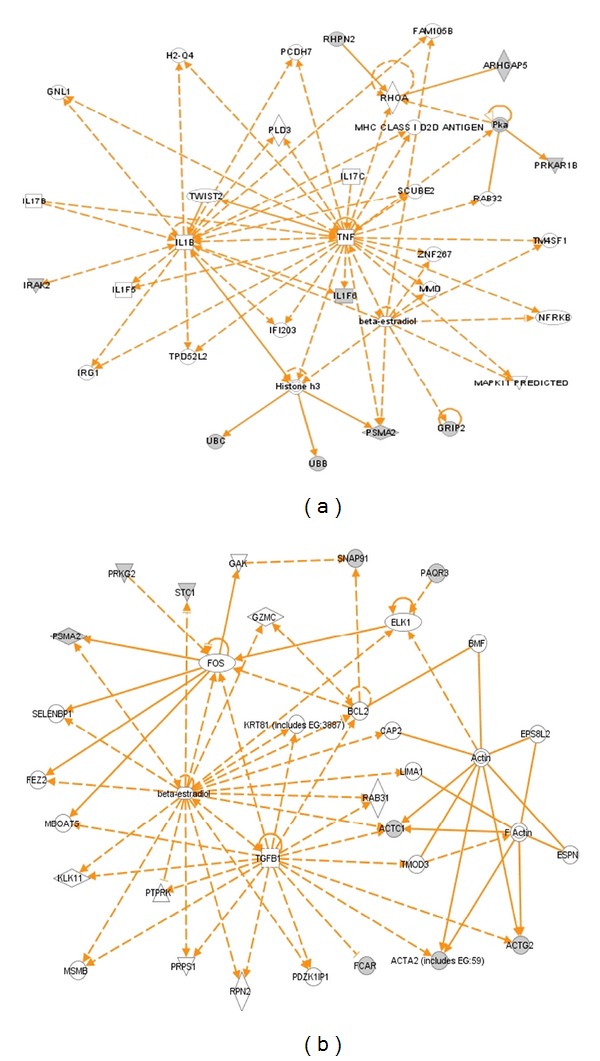
Significant signaling pathway networks mined from nitroproteomic dataset. (a) Network is derived from adenoma nitroproteomic data and function in cancer, cell cycle, reproductive system disease. A gray node denotes an identified nitroprotein or protein that interact with nitroproteins in our study. (b) Network is derived from control nitroproteomic data and function in gene expression, cellular development, and connective tissue development and function. A gray node denotes an identified nitroprotein in our studies. An orange solid edge denotes a direct relationship between two nodes (molecules: proteins, genes). An orange unsolid edge denotes an indirect relationship between two nodes (molecules: proteins, genes). The various shapes of nodes denote the different functions. A curved line means intracellular translocation; a curved arrow means extracellular translocation. Reproduced from Zhan and Desiderio [23], with permission from BioMed Central open access journal, copyright remains with the authors.

The Ingenuity Pathway Analysis (IPA) (http://www.ingenuity.com/) and the MetaCore Pathway Analysis programs (http://www.genego.com/metacore.php/) were used to reveal signaling networks that involve nitroproteins. This method was used to analyze signaling pathway networks that involve nitroproteins identified from human pituitary adenoma [4] and control tissues [3, 16]. For eight nitroproteins from a pituitary control [3, 16] (Table 1), and nine nitroproteins and three non-nitrated proteins that interact with nitroproteins (interactomes) from a pituitary adenoma tissue [4] (Table 1), IPA pathway analyses [23] clearly indicated that those pituitary adenoma nitroproteins and their complexes are involved in the tumor necrosis factor (TNF) and interleukin 1 (IL1) signaling networks (Figure 8(a)), which function in cancer, cell cycle, and reproductive system disease. The nitroproteins in that network include ARHGAP5, PRKAR1B, PSMA2, IL1F6, and RHPN2. The nonnitrated proteins that interact with nitroproteins include IRAK2, GRIP2, and ubiquitin. Three nitroprotein-protein complexes were identified: nitrated proteasome-ubiquitin complex, nitrated beta-subunit of PKA complex, and nitrated IL1F6-IL1 receptor-IL1 receptor-associated kinase-like 2 (IL1F6-IL1R-IRAK2) complex. Those control pituitary nitroproteins are involved in the transforming growth factor beta 1 (TFGB1) and actin cellular skeleton signaling networks (Figure 8(b)), which function in gene expression, cellular development, and connective tissue development. Nitroproteins in that network include SNAP91, FCAR, actin, PRKG2, STC1, PAQR3, and PSMA2. Both networks (Figures 7(a) and 7(b)) include a beta-estradiol signal pathway, which reveals that hormone metabolism is involved in a normal pituitary and pituitary adenoma. It is consistent with the fact that NO participates in pituitary hormone metabolism in normal physiology and tumor interferes with hormone metabolism.

Moreover, among those pituitary adenoma nitroprotein data, twelve statistically significant canonical pathways were identified that involve nitroproteins [23]. The top canonical pathways include p38 MAPK signaling, cell-cycle G2/M DNA damage-checkpoint regulation, the protein-ubiquitination pathway, sonic-hedgehog signaling, GABA-receptor signaling, Toll-like receptor signaling, amyloid processing, the phototransduction pathway, sphingolipid metabolism, IL-10 signaling, hypoxia signaling, LXR/RXR activation, and PXR/RXR activation [23]. Three statistically significant toxicity pathways were mined, including hepatic cholestasis, PXR/RXR activation, and LXR/RXR activation. Among those control pituitary nitroprotein data, twelve statistically significant canonical pathways were identified that involve nitroproteins [23], including clathrin-mediated endocytosis, caveolar-mediated endocytosis, VEGF signaling, regulation of actin-based motility by Rho, Fcy receptor-mediated phagocytosis in NRF2-mediated oxidative-stress response, macrophages and monocytes, tight-junction signaling, leukocyte extravasation signaling, integrin signaling, actin-cytoskeleton signaling, and calcium signaling. No statistically significant toxicity pathways were mined.

Among four signaling pathway network systems (mitochondrial dysfunction, oxidative stress, cell-cycle dysregulation, and MAPK-signaling abnormality) that were discovered from pituitary adenoma mapping data, comparative proteomic data, and nitroproteomic data [23], three signaling pathway network systems (oxidative stress, cell-cycle dysregulation, and MAPK-signaling abnormality) are involved in protein nitration; for example, the NRF2-mediated oxidative-stress response, cell-cycle G2/M DNA damage checkpoint regulation, and p38 MAPK signaling were discovered from pituitary adenoma nitroproteomic data [23]. Therefore, pathway systems analysis revealed that tyrosine nitration plays important roles in the pituitary tumorigenesis.

## 8. Future Perspectives

 To date, the qualitative nitroproteomics based on 2DGE-based nitrotyrosine Western blotting [3, 16] and NTAC enrichment [4] has been used to analyze the presence of nitroproteins in a human pituitary postmortem tissues and in a human pituitary adenoma tissue, respectively. Protein domain and motif analyses [4] have been used to identify the structural or functional domain and motif and to locate nitrotyrosine site into the corresponding domain and motif in a nitroprotein to clarify the functional roles of tyrosine nitration. IPA pathway analysis [23] has been used to identify important pathway networks that are involved in nitroproteins and to address functional roles of nitroproteins from a systematic angle. However, much more research is needed to elucidate the real functional roles of tyrosine nitration in pituitary tumorigenesis.

First, nitroproteomics of single-cell types of a pituitary adenoma will be necessary. The pituitary contains multiple cell types, including GH, PRL, TSH, LH/FSH, and ACTH [66]. Those different cell types of pituitary adenomas could have not only a common mechanism in their formation but also some differences among different cell types of pituitary adenoma. Therefore, it is important to study the same and different differentially expressed nitroproteins among the different cell types of pituitary adenomas and to discover specific nitroprotein biomarkers for pituitary adenomas. Laser capture microdissection (LCM) [67, 68] is a promising and powerful technique to enrich and isolate pure pituitary-cell populations from pituitary adenoma and control pituitary tissue, including somatotrophs, lactotrophs, thyrotrophs, gonadotrophs, and corticotrophs. LCM technique has been optimized [69] to enrich and isolate prolactin cells from postmortem pituitary tissues [70] and prolactinoma tissues [71] for proteomics analysis.

Second, quantitative nitroproteomics among different cell types of pituitary adenoma and controls are needed to determine nitroproteins that are unique to each cell type of pituitary adenoma. With precious LCM-enriched cell types, isobaric tags for relative and absolute quantification- (iTRAQ-) based quantitative proteomics and two-dimensional difference in-gel electrophoresis- (2D-DIGE-) based quantitative proteomics would be the first method to analyze differentially expressed nitroproteins (DENPs) among different cell types of pituitary adenomas. 2D-DIGE in combination with fluorescent dye stains would separate identical proteins from two samples tagged with different fluorescent dyes (Cy3, Cy5) in a single 2D gel and comigration to the same spot position [66, 72]. Quantification of identical proteins from two samples would be determined from the difference in signal intensity of two fluorescent dyes. Compared to classical 2DGE-based comparative proteomics, 2D-DIGE eliminates between-gel variations, improves reproducibility, provides a higher throughput, has a wider dynamic range, and requires only half of the protein-loading amount of conventional 2DGE [66, 73]. Therefore, 2D-DIGE-based quantitative proteomics would especially be suitable for those rare sample sources such as LCM pituitary cells from pituitary adenomas and controls [66]. 

iTRAQ-based quantitative proteomics involves different samples labeled with different iTRAQ reagents, equally mixed for strong cation exchange (SCX), liquid chromatography (LC), and tandem mass spectrometry (MS/MS) analyses. The intensities of reporter ions are used to quantify DENPs. Because iTRAQ equally mixes labeled samples, it will require less amount of each sample, similar to 2D-DIGE. iTRAQ would be suitable for rare samples, especially LCM-enriched samples. Moreover, iTRAQ can overcome the drawbacks of 2D-DIGE, including the limited range of 2D-DIGE to separate proteins (e.g., for a pH 3–10 NL gel, distribution of detected protein in the area of pH 4–8 and mass 15–100 kDa) [66] and the difficulty to detect low-abundance and hydrophobic proteins. However, iTRAQ cannot detect protein isoforms, whereas 2D-DIGE can [66]. Therefore, 2D-DIGE and iTRAQ would be combined to analyze DENPs unique to each cell type of pituitary adenoma to maximize nitroproteome coverage.

Third, tyrosine nitration decreases electron density of the phenolic ring of a tyrosine residue to diminish the interaction intensity between enzyme and substrate or between receptor and ligand. The three-dimensional spatial structure of a protein determines its biological functions. For a pituitary adenoma-related nitroprotein, if its three-dimensional spatial structure can be reconstructed from X-ray crystallography, then it will be very easy to interpret the effect of tyrosine nitration on the 3D structure of a nitroprotein. Meanwhile, based on the 3D structure and tyrosine nitration site and domain, it is possible for one to design a small drug towards the 3D structure and domain that contains tyrosine nitration. For example, sphingosine-1-phosphate lyase 1 (Figure 6(a)) is an enzyme, and two nitrotyrosines were found within the enzymatic activity region. If the 3D structure of sphingosine-1-phosphate lyase 1 could be reconstructed from X-ray crystallography data, then one could clearly interpret the effect of two tyrosine nitrations on its structure and functions.

Finally, a pituitary gland participates in several different hypothalamic-pituitary-target organ axes. A pituitary adenoma would impact those axes systems in a whole-body disease. Therefore, instead of pituitary adenoma tissue, cerebrospinal fluid (CSF) and blood plasma must be studied because some secreted proteins and peptides enter into the CSF and blood circulation in a pituitary adenoma patient [74]. Also, CSF and blood specimens are much more accessible from patients and controls than pituitary tissues and overcome the limitations of pituitary tissues [74]. Quantitative nitroproteomics can detect those nitroproteins and nitropeptides in a patient's CSF and blood plasma. Those CSF and blood plasma nitroproteomic and nitropeptidomic variations would lead to the development of accurate biomarkers for predictive diagnosis, early-stage diagnosis, and measurement of prevention and therapy responses.

## 9. Conclusions

Tyrosine nitration is an important molecular event in pituitary adenoma and is extensively associated with pituitary physiological and pathological processes. 2DGE-based nitrotyrosine Western blot coupled with MS/MS was used to detect 32 nitrotyrosine positive gel spots and eight nitroproteins and modified sites from a pituitary postmortem tissue [3, 16]. NTAC-based enrichment coupled with MS/MS was used to identify nine nitroproteins and modified sites and three nitroprotein-interacting proteins (interactomes) from a pituitary adenoma tissue [4], and most of nitrotyrosine sites were located within important protein domains/motifs. Tyrosine nitration was involved in three pathway network systems (oxidative stress, cell-cycle dysregulation, and the MAPK-signaling abnormality) that are significantly associated with pituitary adenomas [23]. Moreover, MALDI UV laser causes photodecomposition (loss of one or two oxygen atoms) of a nitro group in a nitropeptide. Recognition of the photodecomposition pattern would assist in the interpretation of an MS spectrum of an endogenous nitropeptides. In the future, one needs to perform quantitative nitroproteomics of each cell type of a pituitary adenoma to discover the nitroprotein biomarker unique to each cell type of a pituitary adenoma and further analysis of the 3D structure of that nitroprotein. In addition, it is important to develop quantitative nitroproteomics of body fluids (CSF and blood plasma) of pituitary adenoma patients to recognize body-fluid nitroproteins or nitropeptide patterns.

## Figures and Tables

**Table 1:** Nitroprotein and unnitrated protein identified from pituitary adenoma [4] and control tissue [3, 16].

Protein name	nY site
Pituitary adenoma	

Nitrated protein	
Rho-GTPase-activating 5 [Q13017] (ARHGAP5)	Y^550^
Leukocyte immunoglobulin-like receptor A4 [P59901]	Y^404^
Zinc finger protein 432 [O94892]	Y^41^
PKA-beta regulatory subunit [P31321] (PRKAR1B)	Y^20^
Sphingosine-1-phosphate lyase 1 [O95470]	Y^356^
	Y^366^
Centaurin-beta 1 [Q15027]	Y^485^
Proteasome subunit alpha type 2 [P25787] (PSMA2)	Y^228^
Interleukin 1 family member 6 [Q9UHA7] (IL1F6)	Y^96^
Rhophilin 2 [Q8IUC4] (RHPN2)	Y^258^
Nitroprotein-interacted protein	
Interleukin-1 receptor-associated kinase-like 2 (IRAK-2) [O43187] (IRAK2)	
Glutamate receptor-interacting protein 2 [Q9C0E4] (GRIP2)	
Ubiquitin [P629881] (UBB or UBC)	

Pituitary control	

Nitrated protein	
Synaptosomal-associated protein (SNAP91)	Y^237^
Ig alpha Fc receptor [P24071] (FCAR)	Y^223^
Actin [P03996] (ACTA2, ACTG2, and ACTC1)	Y^296^
PKG 2 [Q13237] (PRKG2)	Y^354^
Mitochondrial cochaperone protein HscB [Q8IVVL3]	Y^128^
Stanniocalcin 1 [P52823] (STC1)	Y^159^
Proteasome subunit alpha type 2 (PSMA2)	Y^228^
Progestin and adipoQ receptor family member III [Q6TCH7] (PAQR3)	Y^33^

nY: nitrotyrosine. Modified from Zhan and Desiderio [3, 4, 16], with permission from Elsevier Science, copyright 2004, 2006, and 2007.

## Abbreviations

ACTH: Adrenocorticotropin
CSF: Cerebrospinal fluid
DENP: Differentially expressed nitroprotein
ELISA: Enzyme-linked immunosorbent assay
eNOS: Endothelial nitric oxide synthase
ESI: Electrospray ionization
FSH: Follicle-stimulating hormone
GH: Growth hormone
IFN: Interferon
IL1: Interleukin 1
iNOS: Inducible nitric oxide synthase
IPA: Ingenuity pathway analysis
iTRAQ: Isobaric tags for relative and absolute quantification
LC: Liquid chromatography
LCM: Lasercapture microdissection
LH: Luteinizing hormone
LPS: Lipopolysaccharide
MALDI: Matrix-assisted laser desorption ionization
Mr: Relative molecular weight
MS: Mass spectrometry
MS/MS: Tandem mass spectrometry
nNOS: Neuronal nitric oxide synthase
NO: Nitric oxide
NOS: Nitric oxide synthase
NTAC: Nitrotyrosine affinity column
pI: Isoelectric point
PKA: cAMP-dependent protein kinase
PRL: Prolactin
RNS: Reactive nitrogen species
ROS: Reactive oxygen species
SCX: Strong cation exchange
TGFB1: Transforming growth factor beta 1
TNF: Tumor necrosis factor
1DGE: One-dimensional gel electrophoresis
2DGE: Two-dimensional gel electrophoresis
2D-DIGE: Two-dimensional differential in-gel electrophoresis.
